# Rapid modulation of synaptogenesis and spinogenesis by 17β-estradiol in primary cortical neurons

**DOI:** 10.3389/fncel.2015.00137

**Published:** 2015-04-14

**Authors:** Katherine J. Sellers, Filippo Erli, Pooja Raval, Iain A. Watson, Ding Chen, Deepak P. Srivastava

**Affiliations:** ^1^Department of Basic and Clinical Neuroscience, Institute of Psychiatry, Psychology and Neuroscience, King’s College LondonLondon, UK; ^2^Department of Biotechnology and Biosciences, Univeristy of Milano-BicoccaMilano, Italy

**Keywords:** 17β-estradiol, PSD-95, dendritic spines, neuroligin-1, estrogen receptor, ERK1/2, Akt, mTOR

## Abstract

In the mammalian forebrain, the majority of excitatory synapses occur on dendritic spines. Changes in the number of these structures is important for brain development, plasticity and the refinement of neuronal circuits. The formation of excitatory synapses involves the coordinated formation of dendritic spines and targeting of multi-protein complexes to nascent connections. Recent studies have demonstrated that the estrogen 17β-estradiol (E2) can rapidly increase the number of dendritic spines, an effect consistent with the ability of E2 to rapidly influence cognitive function. However, the molecular composition of E2-induced spines and whether these protrusions form synaptic connections has not been fully elucidated. Moreover, which estrogen receptor(s) (ER) mediate these spine-morphogenic responses are not clear. Here, we report that acute E2 treatment results in the recruitment of postsynaptic density protein 95 (PSD-95) to novel dendritic spines. In addition neuroligin 1 (Nlg-1) and the NMDA receptor subunit GluN1 are recruited to nascent synapses in cortical neurons. The presence of these synaptic proteins at nascent synapses suggests that the machinery to allow pre- and post-synapses to form connections are present in E2-induced spines. We further demonstrate that E2 treatment results in the rapid and transient activation of extracellular signal-regulated kinase 1/2 (ERK1/2), Akt and the mammalian target of rapamycin (mTOR) signaling pathways. However, only ERK1/2 and Akt are required for E2-mediated spinogenesis. Using synthetic receptor modulators, we further demonstrate that activation of the estrogen receptor beta (ERβ) but not alpha (ERα) mimics rapid E2-induced spinogenesis and synaptogenesis. Taken together these findings suggest that in primary cortical neurons, E2 signaling via ERβ, but not through ERα, is capable of remodeling neuronal circuits by increasing the number of excitatory synapses.

## Introduction

The distribution, number, morphology and strength of synapses along dendrites are crucial determinants of functional neuronal connectivity within brain circuits. In the mammalian forebrain, the majority of excitatory synapses occur on specialized structures known as dendritic spines, which protrude along the length of the dendritic shaft. Functionally, dendritic spines house and compartmentalize the postsynaptic density (PSD), an electron-dense structure containing post-synaptic neurotransmitter receptors, scaffold proteins, and other signaling molecules (Sheng and Kim, 2011). The formation of excitatory synapses requires the coordinated assembly of a large number of protein complexes into the PSD (McAllister, 2007). Many of these proteins are tethered to the PSD though interactions with scaffolding proteins, such as postsynaptic density protein 95 (PSD-95), and play critical roles in synaptic and structural plasticity, glutamate receptor trafficking, and signal transduction (Sheng and Kim, 2011). Recent investigations into the molecular mechanisms of synapse formation have identified several critical events. The recruitment of PSD-95, when part of a protein complex with the trans-synaptic adhesion protein neuroligin-1 (Nlg-1) and N-methyl-D-aspartate receptors (NMDARs), is key for synapse formation (Washbourne et al., 2002; Gerrow et al., 2006; Barrow et al., 2009). Furthermore, Nlg-1 is required for the formation and specification of excitatory synapses (Chubykin et al., 2007; Kwon et al., 2012) while both PSD-95 and Nlg-1 are also required for the activity-dependent stabilization of dendritic filopodia into dendritic spines (Chubykin et al., 2007; Gutiérrez et al., 2009; Kwon et al., 2012). Thus, the presence of these multi-protein complexes at nascent synapses strongly suggests their contributions in the formation of functional connections (McAllister, 2007).

Estrogens, in particular 17β-estradiol (E2), have consistently been shown to regulate and shape synapse structure and function, consistent with the remodeling of neuronal circuitry and the enhancement of cognitive function (Luine and Frankfurt, 2012; Srivastava et al., 2013; Sellers et al., 2015). Interestingly, the cognitive enhancing abilities of E2 are not limited to the classic long-term effects of E2, but can also occur within a rapid (less than 1 h) time frame (Ervin et al., 2013; Srivastava et al., 2013). In addition, activation of the extracellular signal-regulated kinase 1/2 (ERK1/2) and the phoshoinositide 3-kinase (PI3-Kinase) target Akt (also known as protein kinase B, (PKB)) have been shown to be important for the cognitive enhancing abilities of E2 (Fernandez et al., 2008; Fan et al., 2010). More recently it has also been suggested that E2-mediated cognitive enhancements may require local protein synthesis, mediated by the mammalian target of rapamycin (mTOR) kinase pathway (Fortress et al., 2013; Sellers et al., 2015). Consistent with the cognitive enhancing properties, we and others have shown that E2 rapidly increases spine density on cortical and hippocampal neurons within 30 min, suggesting the formation of nascent synaptic connections (Srivastava et al., 2008, 2010; Inagaki et al., 2012). However, whether E2-induced spines are forming synaptic connections, and the precise signaling mechanisms that mediate E2-induced spinogenesis, is currently unclear. Investigations into the estrogen receptors (ERs) that underlie the rapid modulation of synapse density have also identified divergent roles for estrogen receptor alpha (ERα) and beta (ERβ) dependent on the brain region examined (Mukai et al., 2007; Srivastava et al., 2010, 2013). Recent studies have shown that ERβ, but not ERα, can modulate glutamatergic signaling in cortical neurons (Galvin and Ninan, 2014), but which ER is required for E2-mediated spinogenesis in cortical neurons is currently not known.

In this study we have investigated rapid estrogenic-mediated spinogenesis and synaptogenesis. Our data demonstrates that in primary cortical neurons E2 induces the recruitment of the synaptic proteins PSD-95, Nlg-1, and the NMDA receptor subunit GluN1 to synapses within 30 min, indicating that nascent spines were capabel of forming synaptic connections. We further found that E2 rapidly activated ERK1/2, Akt and mTOR kinase pathways, but that only ERK1/2 and Akt pathways were required for E2-induced spinogenesis. Additionally, acute activation of ERβ, but not ERα, was sufficient to increase the density of PSD-95 containing spines in cortical neurons. Taken together, these data suggest that: E2-induced spines are capable of forming synaptic connections; the formation of these nascent connections occurs via the action of specific kinase pathways; and ERβ plays a critical role in mediating the rapid effects of E2 on spinogenesis and synaptogenesis in cortical neurons.

## Methods

### Reagents

Antibodies used: GFP chicken polyclonal (ab13972; Abcam); Phospho-p44/42 MAPK (ERK1/2; Thr202/Tyr204) rabbit monoclonal (D13.14.4E; #4370), ERK1/2 mouse monoclonal (L34F12; #4696), phospho-Akt (Ser473) rabbit monoclonal (D9E; #4060), Akt mouse monoclonal (40D4; #2920), phospho-mTOR (Ser2448) rabbit monoclonal (D9C2; #5536) and mTOR mouse monoclonal (L27D4; #4517) antibodies were all from Cell Signaling Technologies; these antibodies have previously been used to examine rapid steroid signaling in neurons (Srivastava et al., 2005; Briz and Baudry, 2014). GluN1 NMDA receptor subunit mouse monoclonal antibody (556308; BD Biosciences), PSD-95 mouse monoclonal antibody (clone K28/43; 73-028; NeuroMab) and neuroligin1 rabbit polyclonal antibody (H-45; sc-50393; Santa Cruz) have previously been validated and used to examine synaptic localization of these synaptic proteins in primary cortical neurons (Srivastava et al., 2008, 2010; Woolfrey et al., 2009). The ERα (H-184, sc-7207) and ERβ (H-150, sc-8974) rabbit polyclonal antibodies were from Santa Cruz and have previously been used to examine the extranuclear localization of both receptors in neurons (Milner et al., 2005; Dominguez and Micevych, 2010). 17β-estradiol (E8875) was from Sigma and estrogen receptor agonists PPT (1426) and WAY-200070 (3366) were from Tocris. The specificity of WAY-200070 for ERβ has previously been documented: EC50 ERβ: 2.3 nM vs. ERα: 155 nM (Hughes et al., 2008; Liu et al., 2008). The kinase inhibitors U0126 (MEK kinase inhibitor) (9903S) and LY294002 (PI3-Kinase inhibitor) (9901S) were from Cell Signaling Technologies and rapamycin (mTOR inhibitor) (sc-3504) was from Santa Cruz.

### Neuronal Culture and Transfections

Mixed sex cortical neuronal cultures were prepared from Sprague-Dawley rat E18 embryos as described previously (Srivastava et al., 2011). Animals were habituated for 3 days before experimental procedures, which were carried out in accordance with the Home Office Animals (Scientific procedures) Act, United Kingdom, 1986. Cells were plated onto 18 mm glass coverslips (No 1.5; 0117580, Marienfeld-Superior GmbH and Co.), coated with poly-D-lysine (0.2 mg/ml, Sigma), at a density of 3 × 10^5^/well equal to 857/mm^2^. Neurons were cultured in feeding media: neurobasal medium (21103049) supplemented with 2% B27 (17504044), 0.5 mM glutamine (25030024) and 1% penicillin:streptomycin (15070063) (all reagents from Life technologies). Neuron cultures were maintained in presence of 200 μM D, L-amino-phosphonovalerate (D, L-APV, ab120004, Abcam) beginning on DIV (days *in vitro*) 4 in order to maintain neuronal health for long-term culturing and to reduce cell death due to excessive Ca^2+^ cytotoxicity via over-active NMDA receptors (Srivastava et al., 2011). We have previously shown that the presence or absence of APV in the culture media does not affect E2’s ability to increase spine linear density (Srivastava et al., 2008). Half media changes were performed twice weekly until desired age (DIV 23–25). The primary cortical neurons were transfected with eGFP at DIV 23 for 2 days, using Lipofectamine 2000 (11668027, Life Technologies) (Srivastava et al., 2011). Briefly, 4 μg of plasmid DNA was mixed with Lipofectamine 2000 and incubated for 4–12 h, before being replaced with fresh feeding media. Transfections were allowed to proceed for 2 days, which results in approximately 10% transfection efficacy (Xie et al., 2007; Srivastava et al., 2011). Constructs for ERα and ERβ were previously generated and validated: GFP-tagged ERα (Stenoien et al., 2000); V5-tagged ERβ (Wittmann et al., 2007); both constructs were purchased from Addgene.

### Pharmacological Treatments of Neuron Culture

All pharmacological treatments were performed in artificial cerebral spinal fluid (aCSF): (in mM) 125 NaCl, 2.5 KCL, 26.2 NaHCO_3,_ 1 NaH_2_PO_4_, 11 glucose, 5 Hepes, 2.5 CaCl_2,_ 1.25 MgCl_2_, and 0.2 APV). For kinase inhibitor experiments, neurons were pre-treated for 30 min before application of E2 directly over neurons. All compounds were dissolved in either 100% ethanol or DMSO at a concentration of 10 or 1 mM, and serially diluted to a 10X working concentration in aCSF, and applied directly to neuronal cultures. Final concentration of solvent was <0.01%: vehicle control was made up of solvent lacking compound, diluted as test compounds. Treatments were allowed to proceed for indicated time before being lysed for Western blotting or fixed for immunocytochemistry (ICC).

### Immunocytohistochemistry (ICC)

Neurons were washed in PBS and then fixed in either 4% formaldehyde/4% sucrose PBS for 10 min at room temperature followed by incubation in methanol pre-chilled to −20°C for 10 min at 4°C, or in methanol (−20°C) only for 20 min at 4°C. Fixed neurons were then permeabilized and blocked simultaneously (2% Normal Goat Serum, 5425S, Sigma and 0.2% triton x-100) before incubation in primary antibodies overnight and subsequent incubation with secondary antibodies the following day (Srivastava et al., 2011). In the green/purple color scheme, co-localization is indicated by white overlap.

### Quantitative Analysis of Spine Morphologies and Immunofluorescence

Confocal images of double-stained neurons were acquired with a Leica SP-5 confocal microscope using a 63x oil-immersion objective (Leica, N.A. 1.4) as a z-series. Two-dimensional maximum projection reconstructions of images were generated and morphometric analysis (spine number, area and breadth) was done using MetaMorph software (Universal Imaging Corporation) (Srivastava et al., 2011). Morphometric analysis was performed on spines from at least two dendrites (secondary or tertiary branches), totaling 100 μm, from each neuron. Linear density and total gray value of each synaptic protein cluster was measured automatically using MetaMorph (Srivastava et al., 2011). Cultures directly compared were stained simultaneously and imaged with the same acquisition parameters. For each condition, 9–12 neurons from at least 3 separate experiments were used. Experiments were carried out blind to condition and on sister cultures.

### Statistical Analysis

All statistical analysis was performed in GraphPad. Differences in quantitative immunofluorescence, dendritic spine number and morphology were identified by Student’s unpaired *t*-tests. For comparisons between multiple conditions the main effects and simple effects were probed by 1- or 2-way ANOVAs with Tukey correction for multiple comparisons. Error bars represent standard errors of the mean.

## Results

### E2 Increases the Number of PSD-95 Containing Dendritic Spines

**Figure 1:**
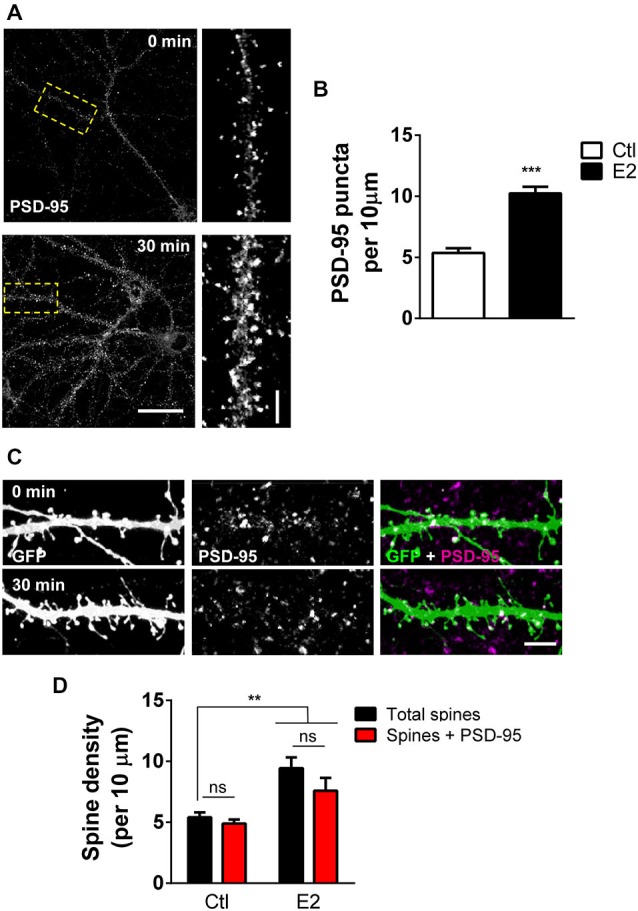
**E2 rapidly recruits PSD-95 to nascent spines. (A)** Primary cortical neurons (DIV 25) processed for PSD-95 immunostaining following treatment with 10 nM E2 or vehicle (30 min). Yellow dashed box denotes section of dendrite magnified in insets. **(B)** Quantification of puncta linear density in neurons from **(A)** reveals an increase in overall PSD-95 expression after E2 treatment (***p < 0.001; *n* = 10–12 cells per condition). **(C,D)** Representative confocal image of dendritic spines on secondary dendrites from eGFP expressing primary cortical neurons (DIV 25) co-stained for PSD-95. Treatment with E2 for 30 min increased the number of dendritic spines containing PSD-95. (**p < 0.01; *n* = 13 cells per condition). Scale bar = 50 μm **(A)**; 5 μm (**A**, inset) and **(C)**.

The scaffolding protein PSD-95 is one of the earliest detectable proteins at the PSD in nascent synaptic connections (Gerrow et al., 2006; McAllister, 2007). It is a critical component of dendritic spines and key for synapse maturation and LTP through its ability to localize several critical synaptic proteins, including neurotransmitter receptors (Sheng and Kim, 2011). As our previous work indicates that E2 can induce the rapid formation of novel dendritic spines (Srivastava et al., 2008), we reasoned that E2-induced spines may also contain PSD-95. To test this we treated DIV 25 primary cortical neurons with 10 nM E2 or vehicle for 30 min: this resulted in an increase in the number of overall PSD-95 puncta (PSD-95 puncta per 10 μm ctl, 5.35 ± 0.39; E2, 10.24 ± 0.54; Figures 1A,B). To determine whether this increase in PSD-95 puncta correlated with an increase in spine linear density, we treated eGFP expressing neurons with E2 for 30 min and double labeled for GFP and PSD-95 (Figure 1C). Consistent with our previous data (Srivastava et al., 2008), E2 significantly increased spine linear density after 30 min (spine density per 10 μm: ctl, 5.4 ± 0.41; E2, 9.4 ± 0.9; Figures 1C,D). A similar increase in PSD-95 containing spines was also seen after E2 treatment: while the linear density of total spines increased by ~57%, an increase of ~61% was seen in the number of PSD-95 containing spines following treatment indicating that E2-induced spines also contained this synaptic protein (Total spines vs. Spines + PSD-95 (per 10 μm): ctl, 5.4 ± 0.41 vs. 4.8 ± 0.34; E2, 9.4 ± 0.9 vs. 7.5 ± 1.04; Figure 1D). Under control conditions approximately 84% of spines were positive for PSD-95, while following E2 treatment ~81% of spines were found to contain PSD-95. Collectively, these data demonstrate that, concurrent with the rapid formation of nascent dendritic spines, E2 treatment results in the recruitment of PSD-95 to newly formed spines.

### Regulation of Post-Synaptic Synaptic Proteins by E2

**Figure 2:**
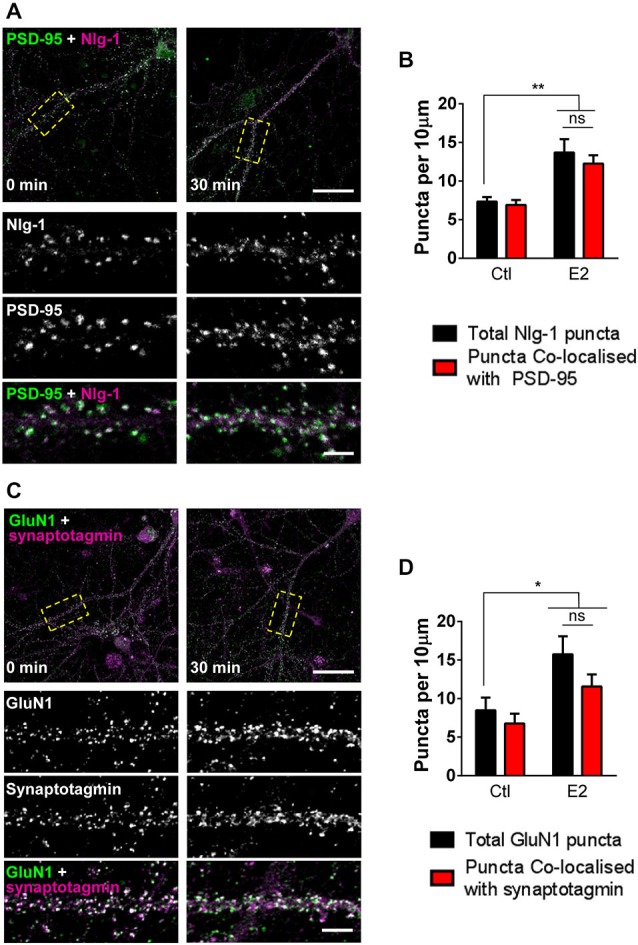
**E2 rapidly increases presence of proteins involved in synaptogenesis at synapses. (A)** Representative confocal images of primary cortical neurons (DIV 25) fixed following treatment with E2 or vehicle (30 min). Neurons were immunostaining for Nlg-1 and PSD-95. Insets are of magnified regions denoted by yellow dashed boxes in low magnification images (top). **(B)** Quantification of **(A)**. Nlg-1 puncta density increases following E2 treatment; a concurrent increase in Nlg-1/PSD-95 co-localized puncta is also observed (**p < 0.01, *n* = 9–10 cells). **(C)** Confocal images of DIV 25 primary cortical neurons treated with or without E2 for 30 min and co-stained for NMDA receptor subunit GluN1 and pre-synaptic protein synaptotagmin. Yellow dashed boxes in low magnification images (top) indicate region of dendrite magnified in insets. **(D)** Quantification of **(C)**. After 30 min of treatment with E2, overall GluN1 puncta density increases; measurements of GluN1/synaptotagmin co-localized puncta also reveal an increase in density (*p < 0.05; *n* = 10 cells per condition). In all images, overlap is indicated by white. Scale bar = 5 μm.

Since E2 rapidly increases the number of PSD-95 containing spines, we reasoned that a similar effect may be seen in synaptic proteins known to interact with this scaffold protein. Nlg-1 belongs to the neuroligin/neurexin family of adhesion proteins and plays an important role in connecting the pre- and post-synaptic membranes (Gerrow et al., 2006; Barrow et al., 2009). Accumulation of pre-formed complexes of Nlg-1 and PSD-95 are thought to be required for the formation of nascent functional connections (Gerrow et al., 2006; Chubykin et al., 2007). To see whether treatment with E2 could also increase the density of Nlg-1/PSD-95 complexes, E2 treated cells were co-stained for Nlg-1 and PSD-95. Under control conditions, >85% of Nlg-1 puncta co-localized with PSD-95, indicating the presence of Nlg-1/PSD-95 complexes. Consistent with an increase in PSD-95 density, following treatment with E2, an increase in the overall number of Nlg-1 puncta was observed (Figures 2A,B). Importantly, the majority of Nlg-1 puncta (~85%) were still co-localized with PSD-95 after E2 treatment indicating that E2 had increased the number of Nlg-1/PSD-95 synaptic complexes (Total Nlg-1 vs. co-localized Nlg-1/PSD-95 puncta (per 10 μm): ctl, 7.3 ± 0.58 vs. 6.5 ± 0.64; E2, 13.7 ± 1.7 vs. 12.2 ± 1.07; Figures 2A,B).

One of the most critical events in synaptogenesis is the recruitment of NMDA receptors to nascent synapses (Washbourne et al., 2002; Barrow et al., 2009). While we have previously shown that E2 can rapidly increase the number of GluN1 NMDA receptor subunits (Srivastava et al., 2008), it was not clear whether this reflected a targeting of the receptor subunit to synaptic or extra-synaptic locations. Thus, to investigate whether E2 treatment resulted in the targeting of GluN1 to synaptic or extra-synaptic locations, treated neurons were co-stained for GluN1 and the pre-synaptic marker synaptotagmin, and assessed for the number of co-localized puncta. Consistent with our previous data, challenge with E2 increases the total number of GluN1 puncta (Figures 2C,D). Under control condition, >75% of GluN1 puncta were found to co-localize with synaptotagmin, indicating a predominance of synaptic GluN1-containing NMDA receptors. Critically, after 30 min of E2 treatment, concomitant with an increase in GluN1 puncta, we found that the majority of these puncta (>73%) remained positive for co-localization with synaptotagmin (Total GluN1 vs. co-localized GluN1/synaptotagmin puntca (per 10 μm): ctl, 8.5 ± 1.65 vs. 6.8 ± 1.26; E2, 15.7 ± 2.34 vs. 11.6 ± 1.55; Figures 2C,D), strongly suggesting that E2-mediated increase in GluN1 puncta were being targeted to synapses. Taken together, these data demonstrate that E2-induced spinogenesis is accompanied by the targeting of key proteins required for synaptogenesis to nascent spines. Thus, E2-induced spines display the hallmarks of being able to form synaptic connections with appropriate pre-synaptic partners.

### E2-Induced Spinogenesis Requires Activation of Distinct Downstream Kinase Pathways

**Figure 3:**
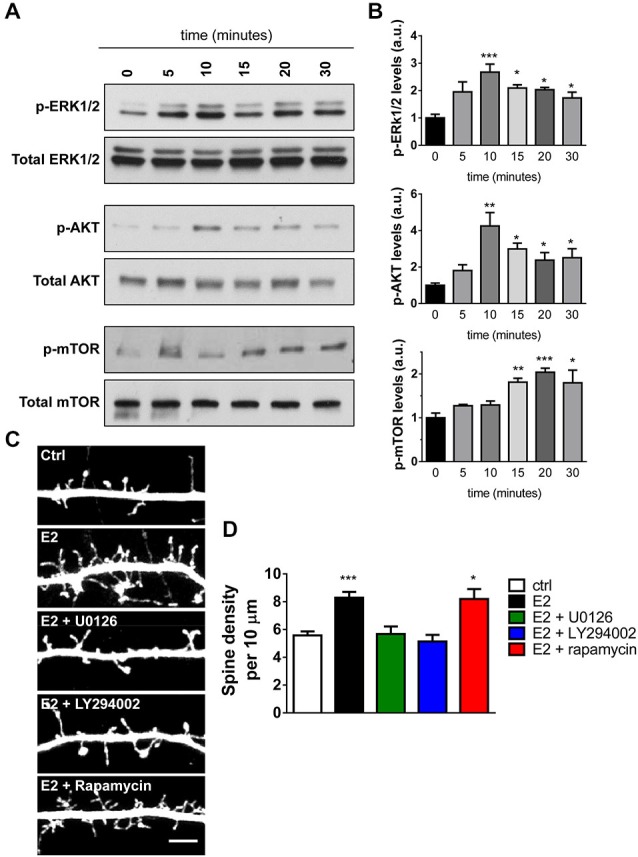
**Activation and requirement of specific kinase pathways during E2-mediated spinogenesis. (A)** Western Blot analysis of ERK1/2, Akt, and mTOR pathways in cell lysates of neurons treated with E2 for increasing amounts of time. Membranes were incubated with phospho-specific antibodies followed by incubation with antibodies against total protein. **(B)** Quantification of the temporal activation of kinase pathways following E2 treatment; all time points were compared to 0 min (untreated) (*p < 0.05; **p < 0.01; ***p < 0.001; *n* = 4 independent experiments). **(C)** Representative confocal images of dendritic spines on secondary dendrites from DIV 25 eGFP expressing primary cortical neurons. Neurons were pretreated (30 min) with vehicle (ctrl) or MEK/ERK1/2 inhibitor U0126, PI3-K/Akt inhibitor LY294002, or mTOR inhibitor rapamycin followed by treatment with vehicle or E2 for an additional 30 min. **(D)** Quantification of linear spine density from cells in **(C)**. Pretreatment with U0126 or LY294002 but not rapamycin blocked E2-mediated spinogenesis (*p < 0.05; ***p < 0.001; *n* = 12–16 cells per condition). Scale bar = 5 μm.

The ability of E2 to induce spinogenesis has been widely reported (Luine and Frankfurt, 2012; Srivastava et al., 2013; Sellers et al., 2015); however, the underlying molecular mechanisms have yet to be fully unraveled. Recent investigations have shown that in the hippocampus, E2 administration results in the rapid and transient activation of ERK1/2, Akt, and degradation of phosphatase and tensin homolog (PTEN), events which preceded the activation of mammalian target of rapamycin (mTOR) and induction of local protein synthesis (Fernandez et al., 2008; Fan et al., 2010; Fortress et al., 2013; Briz and Baudry, 2014). Phosphorylation (activation) of these kinases has previously been implicated in multiple neuronal processes, including spinogenesis (Sweatt, 2004; Jaworski and Sheng, 2006). In addition, activation of these pathways are also critical for E2’s ability to enhance performance on object recognition and object place memory tasks (Fernandez et al., 2008; Fan et al., 2010; Fortress et al., 2013). However, much less is known about which kinase pathways are activated in response to acute E2 treatment in primary cortical neurons and moreover, whether activation of these pathways is required for E2-induced spinogenesis. Therefore, in order to ascertain which kinase pathways were being activated in response to acute E2 treatment, we examined the temporal course of kinase activation by Western Blotting. As previously described (Srivastava et al., 2008) application of E2 resulted in a rapid increase in ERK1/2 phosphorylation (Figure 3A). ERK1/2 activation was maximal after 10 min and decreased over time, yet remained elevated compared to baseline at 30 min (Figure 3B). E2 also induced a similar temporal profile of activation of Akt (Figure 3A); maximal activation of Akt was observed after 10 min and decreased with time but also remained elevated compared to baseline after 30 min (Figure 3B). In contrast, when we examined the phosphorylation of mTOR, we found a delayed and modest activation (Figure 3A); activation of mTOR was not seen until 15 min, peaking at 20 min, and persisted until 30 min (Figure 3B).

We next sought to determine whether activation of these kinase pathways were required for E2-induced spinogenesis. DIV 25 eGFP expressing cortical neurons were pretreated for 30 min with specific kinase inhibitors before challenge with E2 or vehicle for a further 30 min. We used the following kinase pathways inhibitors: the MEK/ERK1/2 pathway inhibitor U0126; LY294002 an inhibitor of the PI3-K/Akt pathway; and the mTOR inhibitor rapamycin. Treatment with the inhibitors alone did not affect spine numbers (data not shown). However, inhibition of the MEK/ERK1/2 and PI3-K/Akt pathways blocked E2-induced spine formation, but inhibition of mTOR had no effect on E2-mediated spinogenesis (spine density per 10 μm: ctrl, 5.5 ± 0.28; E2, 8.3 ± 0.42; E2 + U0126, 5.6 ± 0.54; E2 + LY294002, 5.3 ± 0.31; E2 + rapamycin, 8.8 ± 0.57, Figures 3C,D). Taken together these data demonstrate that E2 is capable of rapidly activating multiple kinase pathways with different temporal dynamics in primary cortical neurons. Moreover, that E2-mediated spinogenesis is dependent on activation of the MEK/ERK1/2 and PI3-K/Akt, but not mTOR, pathways.

### Extranuclear Localization of ERs in Cortical Neurons

**Figure 4:**
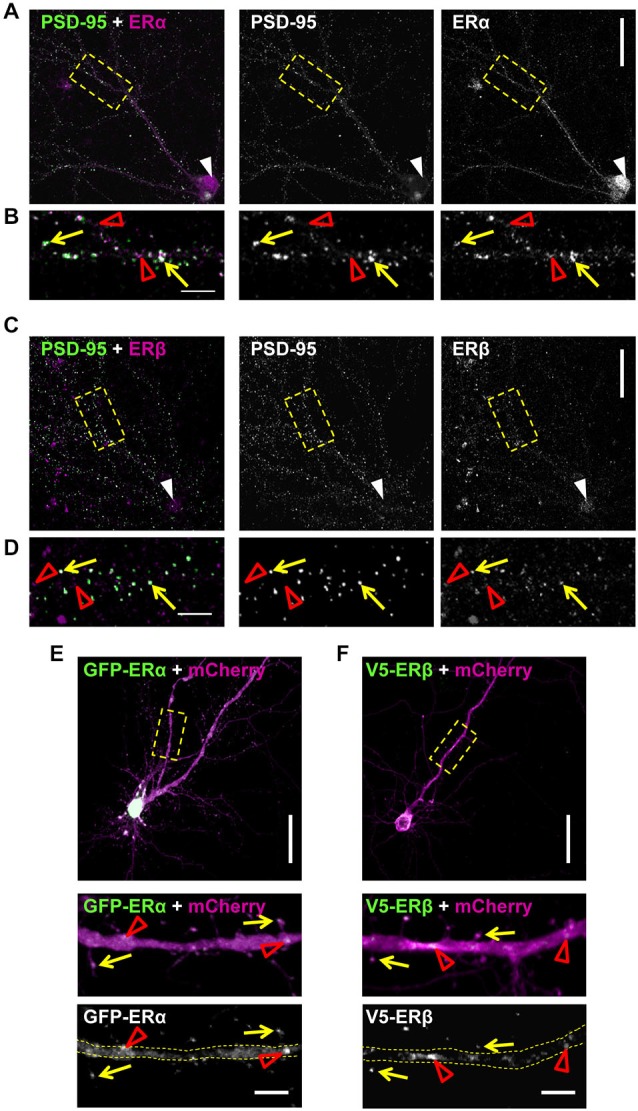
**Extranuclear localization of classic estrogen receptors (ERs) in primary cortical neurons. (A)** Representative confocal images of primary cortical neurons (DIV 23) co-stained for endogenous ERα and PSD-95. Low magnification images demonstrates nuclear staining for ERα (white arrow heads) in addition to presence along dendrites. Yellow dashed boxes indicate magnified dendritic regions shown in **(B)**. **(B)** High magnification images reveal that a subset of ERα puncta co-localizes with PSD-95 (yellow arrows); the remaining puncta are likely present in dendrites (open red arrow heads). **(C)** Co-staining of endogenous ERβ and PSD-95 reveals the presence of the receptor in the nucleus (white arrow head) and along dendrites in DIV 23 primary cortical neurons. **(D)** Examination of distal dendrites as denoted by yellow dashed box in **(C)** reveals that ERβ is present at both extra-synaptic synaptic regions, as demonstrated by co-localization (yellow arrows) or lack thereof (open red arrow heads) with PSD-95. **(E,F)** Representative confocal images of DIV 25 primary cortical neurons ectopically co-expressing GFP-ERα or V5-ERβ with mCherry (to outline neuronal morphology). Yellow dashed boxes denote regions of dendrites magnified in insets. Ectopic ERs localize along dendrites (open red arrow heads) and to dendritic spines (yellow arrows). Scale bar = 50 μm **(A,C,E,F)**; 5 μm (**B,D,E** inset and** F** inset).

The rapid effects of E2 are thought to be mediated by membrane bound/associated ERs (Luine and Frankfurt, 2012; Sellers et al., 2015). Several studies have demonstrated that the classical ERs, ERα and ERβ, have an extranuclear distribution in cortical and hippocampal neurons, with a subset of receptors localizing to synapses (Waters et al., 2011; Almey et al., 2014). In order to determine whether ERα and ERβ had an extranuclear distribution and moreover, localized to synapses in our primary cortical cultures, we immunostained DIV 25 cortical neurons for either ERα or ERβ and PSD-95. As previously described (Almey et al., 2014), both receptors displayed an extranuclear localization in primary cortical neurons (Figures 4A,B). The specificity of staining was determined by no primary antibody controls (data not shown). Confocal imaging of endogenous ERα revealed that the receptor was localized in the nucleus (white arrow head) and along dendrites (Figure 4A). When distal dendrites were examined, ERα was found to be present in punctate structures along dendrites (Figure 4B; open red arrow heads) with a subset of ERα puncta co-localizing with PSD-95 (Figure 4B; yellow arrows). Similarly, endogenous ERβ was present in the nucleus (white arrow heads) and in punctate structures along the dendrite (Figure 4C). ERβ puncta were also observed along distal dendrites (Figure 4D; red open arrow heads) where a subset of puncta co-localized with PSD-95 (Figure 4D; yellow arrows). However, puncta for both receptors that did not co-localize with PSD-95 were also observed, suggesting that these receptors are additionally present at extrasynaptic regions, potentially along dendrites (Figures 4B,C, open red arrow heads). This subcellular distribution is consistent with the receptors being able to engage with the machinery required for the formation of dendritic spines.

Recently the specificity of estrogen receptor antibodies have been called into question (Snyder et al., 2010). Therefore, in order to confirm the post-synaptic localization of ERα and ERβ, we ectopically expressed GFP-tagged ERα or V5-tagged ERβ constructs in DIV 25 cortical neurons (Figures 4E,F). Co-expression of GFP-ERα with mCherry, as a morphological marker, demonstrated that ectopic ERα localized along dendrites (open red arrow heads) and in dendritic spines (Figure 4E; yellow arrows). In a similar manner, co-expression of V5-ERβ with mCherry also revealed immunoreactive staining for the receptor along dendrites (open red arrow heads) and within a subset of dendritic spines also being positive for ERβ (Figure 4F; yellow arrows). Collectively, these data suggest that the classic ERs, ERα and ERβ, have an extranuclear distribution in cortical neurons and furthermore, are present at a subset of synapses and dendritic spines, locating them at an ideal site in which to mediate rapid estrogenic-mediated spinogenesis and synaptogenesis.

### Regulation of Rapid Spinogenesis and Synaptogenesis by Distinct ERs

**Figure 5:**
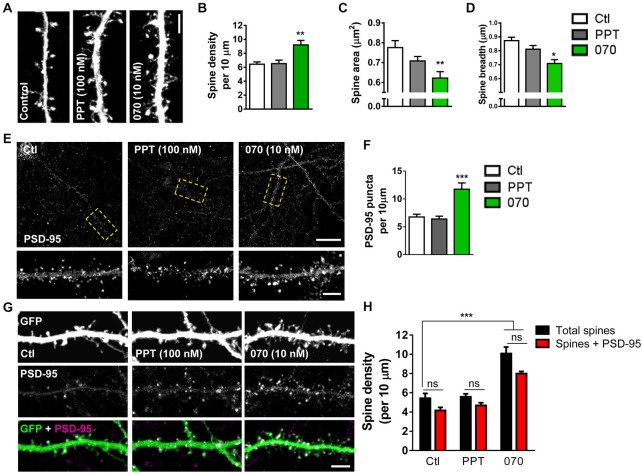
**Acute activation of ERβ but not ERα induces formation of PSD-95 containing dendritic spines**. **(A)** Representative images of dendritic spines on secondary dendrites of eGFP expressing primary cortical neurons (DIV 25) following treatment with PPT (100 nM), WAY-200070 (070; 10 nM) or vehicle (ctl) for 30 min. **(B)** Quantification of **(A)**. Treatment with 070, but not PPT increased spine linear density (**p < 0.01; *n* = 11–15 cells per condition). **(C,D)** Detailed analysis of dendritic spine morphology in response to synthetic ER agonist treatment; 070 leads to decrease in overall spine area and breadth; PPT treatment has no effect on spine morphology (spine area **p < 0.01; spine breadth *p < 0.05; *n* = 11–15 cells per condition). **(E,F)** Confocal images of primary cortical neurons (DIV 25) immunostained for PSD-95; puncta linear density increased with treatment 070, but not PPT (***p < 0.001; *n* = 12 cells per condition). **(G,H)** Representative images of dendritic spines on eGFP expressing primary cortical neurons (DIV 25), co-stained for PSD-95, and quantified for number of PSD-95 containing dendritic spines following application of ER agonists. Activation of ERβ (070) but not ERα (PPT) for 30 min resulted in an elevation of dendritic spines, and PSD-95 containing spine linear density (***p < 0.001 *n* = 15 cells per condition). Scale bar = 50 μm **(E)**; 5 μm (**A,E** inset, and **G**).

Previous studies have indicated divergent roles of ERα and ERβ in E2-mediated spinogenesis. In hippocampal neurons, activation of ERα, but not ERβ has been shown to result in an increase in spine linear density (Mukai et al., 2007). In contrast, activation of ERβ has been shown to increase spine density in cortical neurons (Srivastava et al., 2010). However, a direct comparison of the contribution of ERα and ERβ to E2-mediated spinogenesis in cortical neurons has not been performed. As our data suggests that both ERα and ERβ are ideally localized to regulate spine-morphogenic actions, we investigated the effects of acutely activating ERα or ERβ using specific agonists, on spine linear density. To evaluate whether signaling through either ER could induce spinogenesis within a rapid time-frame, eGFP-expressing cortical neurons were treated with 100 nM PPT, 10 nM WAY-200070 (070), agonists for ERα or ERβ respectively, or vehicle for 30 min. Treatment with PPT did not significantly alter the number of spines compared to controls within 30 min (Figures 5A,B). Conversely, treatment with 070 for 30 min significantly increased spine linear density, accordant with previous findings (spine density per 10 μm: Ctl, 5.8 ± 0.49; PPT, 5.6 ± 0.31; 070, 10.1 ± 0.68; Figures 5A,B) (Srivastava et al., 2010). A detailed analysis of dendritic spine morphology revealed that treatment with 070 resulted in spines with a smaller area (spine area (μm^2^): Ctl, 0.77 ± 0.035; PPT, 0.7 ± 0.035; 070, 0.62 ± 0.032; Figure 5C). This reduction was reflected by a reduction in the average breadth of spines (spine breadth (μm): Ctl, 0.87 ± 0.024; PPT, 0.81 ± 0.026; 070, 0.70 ± 0.027; Figure 5D); treatment with PPT had no effect on either spine area or spine breadth. Taken together, these data demonstrate that in primary cortical neurons activation of ERβ, but not ERα, results in an increase in thin-like dendritic spines.

As E2 treatment induced an increase in the number of spines containing PSD-95 (Figure 1) we next asked whether activation of specific ERs could also recruit this scaffold proteins to nascent synapses. Examination of total PSD-95 puncta revealed that treatment with 070, but not PPT, increased the linear density of PSD-95 puncta within 30 min (PSD-95 puntca (per 10 μm): Ctl, 6.7 ± 0.49; 30 min, PPT, 6.3 ± 0.0.51; 070, 11.7 ± 1.13; Figures 5E,F). We next examined whether ERβ-induced spines also contained PSD-95 by quantifying the number of PSD-95 positive spines. As seen before, under control conditions, the majority of (>75%) spines were positive for PSD-95 (Figures 5G,H). Treatment with PPT did not alter the density of spines containing PSD-95 compared to control. However, treatment with 070 resulted in an increase in the number of spines containing PSD-95 paralleling the effects of E2 on PSD-95 containing spines (Total spines vs. Spines + PSD-95 (per 10 μm): Ctl, 5.4 ± 0.39 vs. 4.2 ± 0.32; PPT, 5.5 ± 0.28 vs. 4.7 ± 0.31; 070, 10.2 ± 0.59 vs. 7.9 ± 0.22; Figures 5G,H). Collectively, these data suggest that ERβ, but not ERα, is required for rapid estrogenic-mediated spinogenesis and synaptogenesis.

## Discussion

Recent studies have begun to uncover the molecular mechanisms underlying the rapid formation of dendritic spines by estrogens (Luine and Frankfurt, 2012; Srivastava et al., 2013; Sellers et al., 2015). However, it is unclear whether E2-induced spines on cortical neurons form synaptic connections, and which ER(s) are responsible for mediating these morphogenic effects. Here we show that, concurrent with the rapid induction of spinogenesis in cortical neurons by E2, there is also a recruitment of synaptic proteins, which are required for the formation excitatory synapses, to nascent dendritic spines. Specifically we find an increase in the density of PSD-95 positive spines concomitantly with a recruitment of Nlg-1 and GluN1 to synapses following acute E2 treatment. Furthermore, we find that while E2 rapidly phosphorylates ERK1/2, Akt and mTOR, only ERK1/2 and Akt pathways are required for E2-mediated spinogenesis. In addition, by using synthetic ER agonists we find that activation of ERβ alone is capable of inducing rapid spinogenesis and recruitment of PSD-95 to newly formed spines. Collectivity these data suggest that E2, signaling through ERβ via ERK1/2 and Akt downstream pathways, increases the number of dendritic spines that contain the molecular machinery required for synaptogenesis. This modulation of synaptic connectivity may contribute to rapid estrogenic remodeling of neuronal circuits and modulation of cognitive function.

While E2 has repeatedly been shown to rapidly increase the number of dendritic spines in both cortical and hippocampal neurons (Mukai et al., 2007; Srivastava et al., 2008), these studies did not demonstrate whether these nascent protrusions contained the molecular machinery required for forming synaptic connections. It is widely accepted that PSD-95 is centrally involved in multiple aspects of synaptic function. PSD-95 has been shown to be necessary for synapse formation (Gerrow et al., 2006) owing to its ability to bind, tether, or stabilize various membrane proteins and signaling molecules in the PSD (McAllister, 2007; Sheng and Kim, 2011). Our data demonstrates that following acute E2 treatment there is an increase in the number of PSD-95 containing dendritic spines, suggesting that E2-induced spines contain the molecular framework for the presence of a PSD. Furthermore it provides the necessary scaffold for the recruitment or stabilization of proteins that are also involved in synaptogenesis at nascent synapses. Consistent with this, we observed an increase in the number of Nlg-1/PSD-95 puncta following E2 treatment. At synapses Nlg-1 anchors both sides of the synapse together by forming trans-synaptic connections with pre-synaptic neurexins (Chubykin et al., 2007; Barrow et al., 2009). Thus, the observed increase in Nlg-1 puncta at synapses, as determined by overlapping localization with PSD-95 following treatment, suggests that E2-induced spines can form physical connections with pre-synaptic termini.

Recent studies have suggested that Nlg-1 is required for the formation and stabilization of nascent connections through the recruitment of PSD-95 and NMDA receptors (Chubykin et al., 2007; Barrow et al., 2009; Gutiérrez et al., 2009; Kwon et al., 2012). Previously we have shown that E2 rapidly increases the density of the NMDA receptor subunit GluN1 (Srivastava et al., 2008). In the present study we demonstrate that GluN1 is indeed recruited specifically to synapses, consistent with increased synaptic Nlg-1. The presence of GluN1 at synapses is also in agreement with the formation of synaptic connections, and based on previous studies, it is likely that GluN1 is recruited to nascent synapses by PSD-95/Nlg-1 complexes (Barrow et al., 2009; Gutiérrez et al., 2009). An increase of synaptic GluN1-containing NMDA receptors suggests that it is well positioned to respond to subsequent activity-dependent stimuli (Xie et al., 2007). Furthermore, the importance of increased localization of GluN1 to synaptic as opposed to extrasynaptic regions, is demonstrated by the role extrasynaptic NMDA receptors play in mediating excitotoxicity signals (Paoletti et al., 2013).

There is a growing appreciation of the complex manner by which estrogens may influence multiple aspects of brain function. E2 has been shown to rapidly activate the ERK1/2, Akt and mTOR pathways (Briz and Baudry, 2014) highlighting the fact that estrogens can rapidly modulate multiple functions via multiple molecular mechanisms in the CNS (McEwen and Alves, 1999). Interestingly, recent studies have shown that the rapid activation of both ERK1/2 and Akt pathways are required for E2 enhancement of performance in object recognition tasks (Fernandez et al., 2008; Fan et al., 2010). Similarly E2 has been suggested to enhance performance in object recognition tasks through local protein synthesis pathways, mediated by the activation of an mTOR pathway (Fortress et al., 2013). Data from the present study demonstrates that E2-mediated spinogenesis in primary cortical neurons requires activation of the ERK1/2 and Akt, but not mTOR pathways. This suggests that the initial formation of nascent spines does not require protein synthesis; it is likely that the ERK1/2 and Akt pathways regulate local signaling. These results complement our previous data, which demonstrates that E2 rapidly increases spine density even in the presence of cycloheximide; a protein synthesis inhibitor (Srivastava et al., 2008). However, it is yet to be determined whether the induction of a local protein synthesis mechanism, as mediated by an mTOR pathway, is required for the stabilization of a nascent synaptic connection and thus consolidation of memory (Srivastava et al., 2013; Sellers et al., 2015). The results in this study further add to well documented findings that suggest signaling via specific ERs have substantial effects on cognitive processes (Luine and Frankfurt, 2012; Ervin et al., 2013; Srivastava et al., 2013), and can specifically modulate spine number via distinct receptors depending on brain regions (Mukai et al., 2007; Srivastava et al., 2010).

Previous studies have demonstrated that E2 increases spine density on both cortical neurons of female rats within 30 min (Inagaki et al., 2012) and CA1 neurons of male rats within a similar time frame (Mukai et al., 2007; Inagaki et al., 2012). Our data correlates with these *in vivo* and *ex vivo* observations. Moreover, the reported increase in spine density on cortical neurons was seen concurrently with enhanced performance in a number of behavioral tasks (Inagaki et al., 2012), whilst the increase in spine density in CA1 neurons was seen in addition to enhanced synaptic plasticity (Mukai et al., 2007). These observations, thus provide a link between E2-driven changes in synapse number with both synaptic function and cognition. Another important consideration is the physiological relevance of the concentration of E2 used in our study. Levels of plasma E2 have been reported to be in the lower picomolar range (Ishii et al., 2007; Hojo et al., 2009; Konkle and McCarthy, 2011), a concentration that some have argued is not sufficient to elicit rapid estrogenic signaling (Balthazart and Ball, 2006; Srivastava et al., 2013). However, several studies have now measured higher pico- to nanomolar concentrations of E2 within specific brain regions, including the cortex (Ishii et al., 2007; Hojo et al., 2009; Konkle and McCarthy, 2011). Indeed, E2 has been previously demonstrated to be rapidly synthesized through the conversion of androgens into estrogens by the enzyme aromatase in brain tissue within 30 min, and may even reach concentrations of 10 nM (Ishii et al., 2007). This high concentration of E2 is sufficient to trigger a rapid cellular response. High pico and nanomolar E2 concentrations have been reported in both male and female brain tissue (Ishii et al., 2007; Hojo et al., 2009; Konkle and McCarthy, 2011) suggesting that E2 may elicit rapid responses in both sexes. It is worth acknowledging that in the current study we have used mixed sex primary neurons, and thus it is not possible to determine whether the reported effects are sex specific. Nevertheless, E2 has been reported to rapidly modulate spine density in both male and females (Mukai et al., 2007; Inagaki et al., 2012), although this may occur in a sexually dimorphic manner (Brandt et al., 2013).

In addition to demonstrating the rapid effects of E2 on spinogenesis and synaptogenesis, we have also established that ERβ is present at synapses in primary cortical neurons and is required for the rapid induction of spinogenesis. Although we find that ERα is also present at synapses, activation of this receptor did not alter spine density. Whether ERα is required for the subsequent stabilization of newly formed spines, or the generation of nascent connections at a different time point in primary cortical neurons, has yet to be determined. We are also unable able to ascertain in this study whether ERα acts in a sex specific manner. Nevertheless we have further demonstrated that the acute activation of ERβ also results in the recruitment of PSD-95 to nascent spines, indicating that ERβ-induced spines are capable of forming synaptic connections. Interestingly these data correspond with recent evidence that demonstrate signaling through ERβ, and not ERα, can rapidly influence glutamatergic signaling and synaptic transmission (Liu et al., 2008; Kramár et al., 2009; Galvin and Ninan, 2014). These data suggest that ERβ has a predominate role in regulating both structural and functional plasticity in cortical neurons. However, recent reports indicate that GPER1 (formally known as GPR30), an estrogen responsive G-protein coupled receptor, is present in cortical neurons and has morphogenic properties (Srivastava and Evans, 2013; Almey et al., 2014). Therefore future studies are required to determine the contribution of this receptor to the regulation of synapse formation in cortical neurons to fully understand the distinct role of each ER in this process.

Data from this study strengthens suggestions that in addition to rapidly regulating synaptic transmission, estrogens also regulate the number of physical connections between neurons. There is an increasing body of evidence suggesting that estrogens are a successful adjunct treatment for a variety of neuropsychiatric disorders, including schizophrenia, depression, and anxiety (Hughes et al., 2009; Torrey and Davis, 2012; Kulkarni et al., 2014). A common hallmark of these disorders is a loss of synaptic connectivity (Penzes et al., 2011). It has been suggested that either targeting mechanisms that modulate glutamatergic signaling or that restore lost connectivity may be a viable therapeutic avenue in the treatment of various neurological diseases including neuropsychiatric disorders (Gray and Roth, 2007; Penzes et al., 2011; Paoletti et al., 2013). It is tempting to hypothesize that some of the benefits seen after estrogen administration may be due to regulation of dendritic spine remodeling (Srivastava et al., 2013; Sellers et al., 2015). However it is essential to determine the molecular underpinnings and the basis by which estrogens are beneficial in these disorders to fully determine the potential for estrogen-based therapies. As such, understanding the pathways and the receptors that underlie the effects of estrogen on dendritic spine plasticity may offer insights into novel therapeutic targets for treatments of many neuropsychiatric disorders (Hughes et al., 2009; Srivastava et al., 2013; Sellers et al., 2015).

## Author and Contributors

KJS, FE, PR, IAW, DC and DPS carried out and performed quantification on all experiments; KJS and DPS. wrote the paper; DPS directed the study.

## Conflict of Interest Statement

The authors declare that the research was conducted in the absence of any commercial or financial relationships that could be construed as a potential conflict of interest.

## Abbreviations

PSD-95: postsynaptic density protein 95
E2: 17β-estradiol
ER*α*: estrogen receptor alpha
ERβ: estrogen receptor beta
PPT: 4, 4′, 4″ -(4-Propyl-[1*H*]-pyrazole-1, 3, 5-triyl) *tris*phenol
WAY-200070 (070): 7-Bromo-2-(4-hydroxyphenyl)-1, 3-benzoxazol-5-ol
NMDAR: N-methyl-D-aspartate receptor
ERK: extracellular signal-regulated kinase
PKB: protein kinase B
PTEN: degradation of phosphatase and tensin homolog
mTOR: mammalian target of rapamycin (mTOR).
